# Peritoneal Carcinoma Unveiling a Hidden Threat: A Case of Malignant Pericardial Effusion

**DOI:** 10.7759/cureus.46059

**Published:** 2023-09-27

**Authors:** Mohammed Abusuliman, Amr M Mohamed, Anas Mahmoud, Tala Beilani, Ibrahim M Ismail-Sayed

**Affiliations:** 1 Internal Medicine, Henry Ford Health System, Detroit, USA; 2 Pediatrics, Faculty of Medicine, Alexandria University, Alexandria, EGY; 3 Internal Medicine, St. Joseph's University Medical Center, Paterson, USA; 4 Medicine, Kansas City University, Kansas City, USA; 5 Critical Care, St. Luke's University Health Network, Bethlehem, USA

**Keywords:** cardiac tamponade, malignant pericardial effusion, pericardiocentesis, peritoneal carcinoma, pocus

## Abstract

Malignant pericardial effusion (MPE) is a slowly progressive and potentially clinically silent condition. Pericardial effusion can arise in oncology patients due to several factors, including disease spreading directly or metastatically, anticancer therapy side effects, or both. Solid and hematological malignancy metastasis more frequently involves the pericardium than primary tumors, with lung cancer being the most common metastatic tumor to involve the pericardium. While 5%-20% of all patients with metastatic neoplasms have pericardial involvement, MPE rarely appears with hemodynamic instability. Occasionally, MPE constitutes the initial manifestation of an underlying malignancy. Diagnosis and treatment require a multidisciplinary approach and a high degree of clinical suspicion.

We present a case of a 59-year-old female with a history of peritoneal carcinoma who presented with persistent dyspnea on exertion following an episode of pneumonia that was treated with antibiotics. Physical examination and bedside point-of-care ultrasound (POCUS) revealed fluid in the pericardial sac. The cytological examination of the fluid revealed it to be of malignant origin, resulting from metastasis from gynecologic adenocarcinoma. Pericardiocentesis was done, and symptoms improved after fluid drainage.

## Introduction

The pericardium is a fibroelastic sac that surrounds the heart; it comprises two layers: fibrous and serous. Typically, there is a small amount of serous fluid (15-50 mL) between the two layers of the pericardium. This fluid acts as a lubricant to facilitate the movement of the heart. Pericardial effusion occurs when the amount of fluid in the pericardial sac exceeds the normal level, which can result in the compression of the heart. The accumulated fluid can be transudate, exudate, or sanguineous [1].

There are various established etiologies of pericardial effusion, which can be classified into several groups [2]: infection, which can be viral, bacterial, fungal, mycobacterial, and parasitic; malignancy, secondary tumors, e.g., lung cancer, that more commonly cause pericardial effusion, and primary tumors such as pericardial mesothelioma that can also lead to pericardial effusion; collagen vascular diseases, autoimmune and rheumatologic diseases that can cause pericardial effusion, e.g., rheumatoid arthritis (RA), systemic lupus erythematosus (SLE), and familial Mediterranean fever (FMF); cardiac, post myocardial infarction pericardial effusion, congestive heart failure (CHF), and cardiac wall rupture and also aortic dissection that is a well-recognized cause of cardiac tamponade; trauma, traumatic injury to the heart, great vessels, and coronaries that can cause bloody pericardial effusion; metabolic, such as uremia, ovarian hyperstimulation, and hypothyroidism; drugs, certain drugs that can cause pericardial effusion such as hydralazine, phenytoin, and chemotherapy drugs, e.g., doxorubicin and cyclophosphamide; radiation, irradiation that can cause pericardial effusion within a mean latency period of one year; and idiopathic, the collection of pericardial fluid that persists for more than three months and has no apparent cause.

All causes of pericardial effusion can lead to cardiac tamponade when sufficient fluid accumulates in the pericardium to compress the heart; on the other hand, when the fluid builds up rapidly, compression can occur with much smaller volumes. Cardiac tamponade is a pericardial syndrome characterized by the hindrance of the diastolic filling of the ventricles, hence reducing the cardiac output. If left untreated, it can lead to cardiogenic shock and produce signs and symptoms of cardiac arrest. It is a life-threatening emergency that is usually associated with chest pain, tachypnea, and dyspnea. Depending on the size and onset, it might be acute, subacute, or chronic. Physical examination signs include jugular vein distention (JVD), muffled heart sounds, and pulsus paradoxus. Hypotension, narrow pulse pressure, and tachycardia can also be detected during physical examination [3].

When a sufficient volume of fluid fills the pericardium rapidly, the heart chambers get compressed, and tamponade develops quickly with lower volumes, classically evident by traumatic heart injury with hemopericardium. The heart chambers are unable to relax as a result of increased pressure, which results in reduced venous return, ventricular filling, and cardiac output [4]. Slow-growing effusions, such as those caused by neoplasms or autoimmune illness, allow for the stretching of the pericardium, allowing for higher amounts to be accommodated, and effusions can reach fairly large amounts before leading to tamponade physiology. In brief, traumatic causes require smaller amounts of fluid to generate hemodynamic instability than medical etiologies such as malignancy, which can cause enormous volumes of fluid to build up in the pericardium before the patients become symptomatic [5].

In this case report, we highlight malignant pericardial effusion (MPE) and tamponade. This case report was presented as an abstract at the American Thoracic Society (ATS) Conference in 2023.

## Case presentation

**Figure 1:**
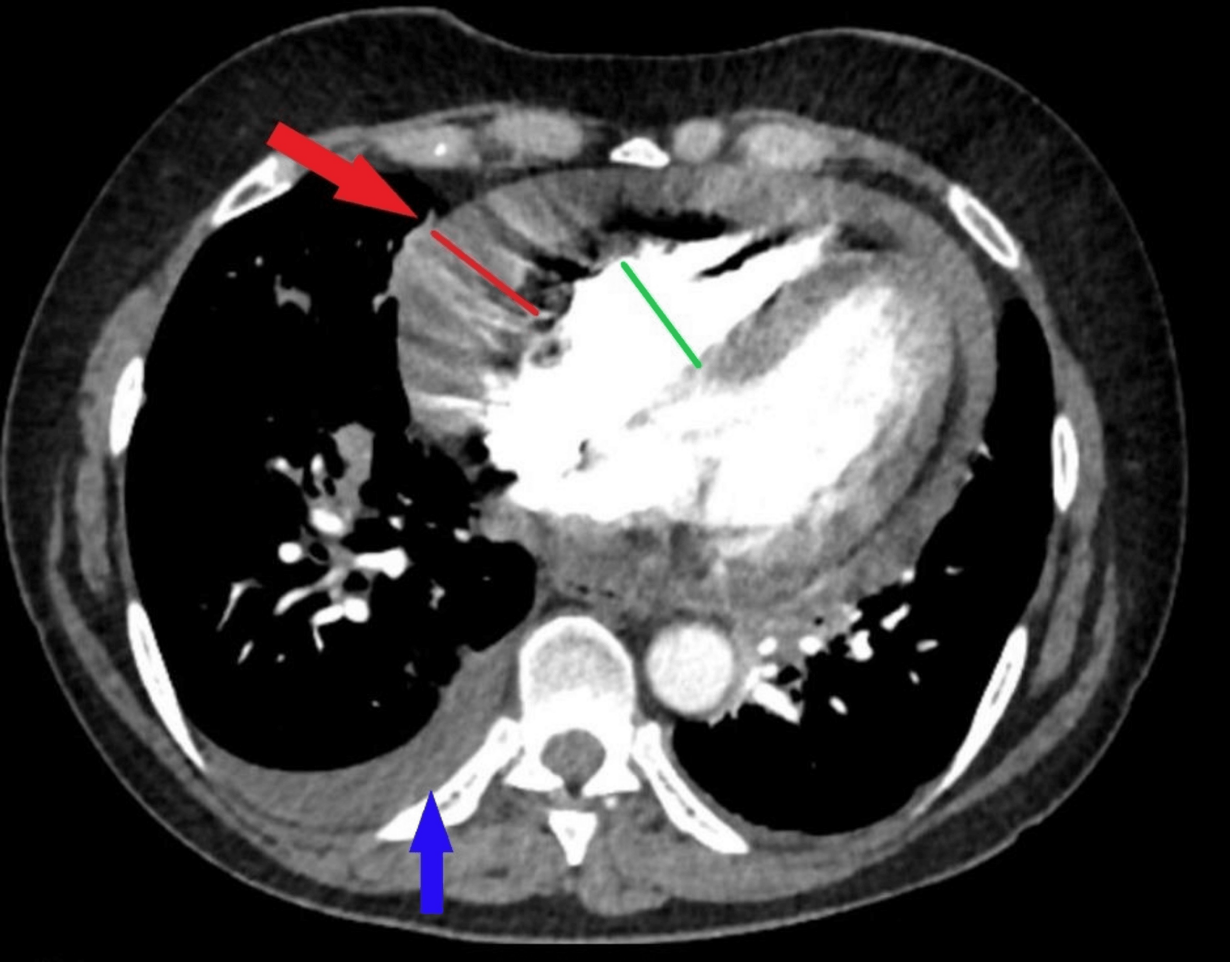
CT angiography study of the chest revealing a moderate to large pericardial effusion (red arrow and line), right-side pleural effusion (blue arrow), and collapsed right ventricle (green line) concerning for a tamponade.

A 59-year-old female with a history of stage IIIC peritoneal carcinoma status post (s/p) total abdominal hysterectomy with bilateral salpingo-oophorectomy (TAH/BSO) with debulking presented to the pulmonary clinic with persistent dyspnea and chest heaviness on exertion. One month prior, she was seen in the emergency department (ED) for a nonproductive cough, nasal congestion, and postnasal drip; a chest X-ray showed multifocal opacities with concerning pneumonia. She was treated and improved with a one-week course of antibiotics; however, she continued to have persistent dyspnea on exertion. On examination, the lungs were clear, but heart sounds were distant. A six-minute walk test showed new exertional hypoxemia, requiring 2 L supplemental O_2_ to end the test with oxygen saturation (SpO_2_) at 92%, and the total distance walked was about 400 meters. Upon reviewing old chest images from one year before, she was noted to have multiple pulmonary nodules of 4-5 mm. At that time, she declined further chemotherapy after only two sessions. Due to the history of malignancy, a CT angiography of the chest was ordered and showed no pulmonary embolism; however, it revealed a moderate to large pericardial effusion with reflux of the contrast into the hepatic veins, right-side pleural effusion, and collapsed right ventricle (RV) concerning for a tamponade (Figure 1).

**Figure 2:**
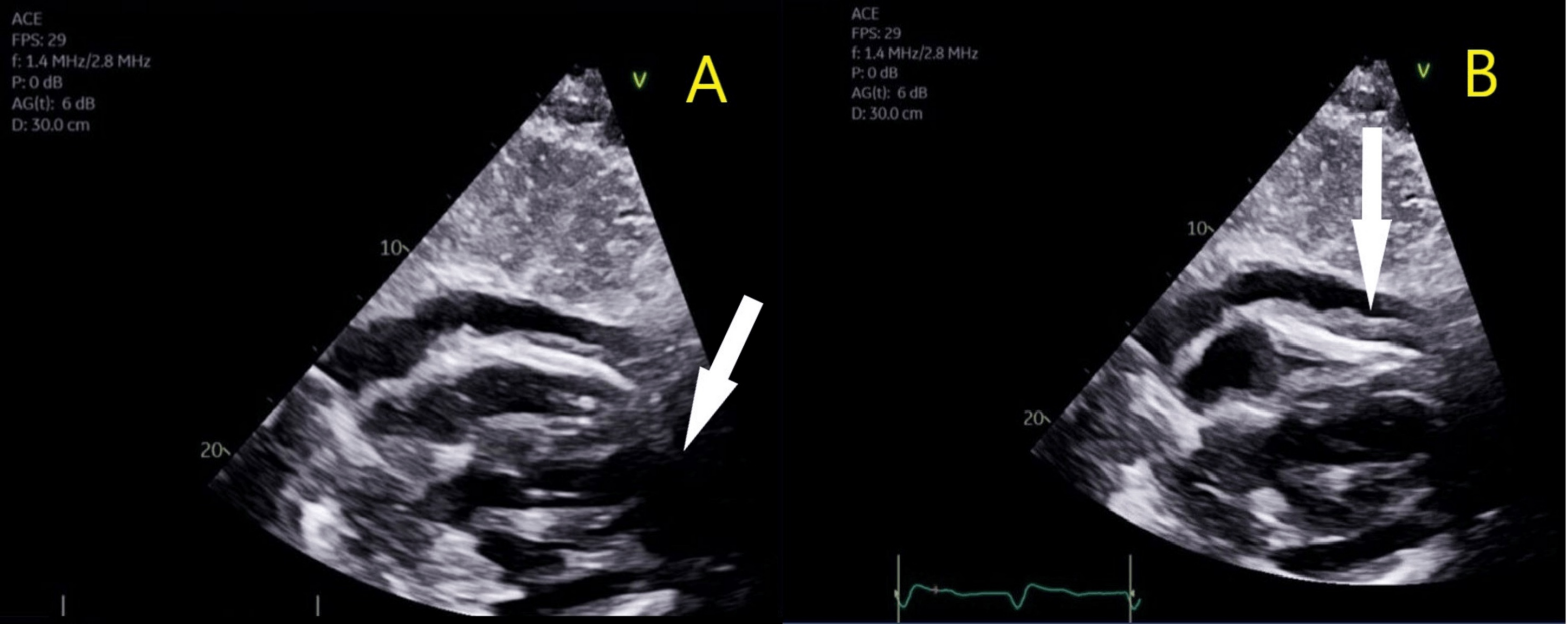
Bedside POCUS showing a large pericardial effusion on apical/subcostal views (panel A, white arrow) and RV collapse during diastole (panel B, white arrow). POCUS, point-of-care ultrasound; RV, right ventricle

The patient was immediately sent to the ED; a bedside point-of-care ultrasound (POCUS) showed a large pericardial effusion on apical/subcostal views and RV collapse during diastole (Figure 2).

The patient remained hemodynamically stable and asymptomatic on room air at rest. An urgent pericardial window drained 550 cc of bloody fluid initially, and the pericardial drain was left for four days. Fluid cytology was consistent with malignant effusion and with metastatic gynecologic primary adenocarcinoma.

The patient had a significant improvement in symptoms after the drainage of pericardial fluid, with an improvement of pleural effusion as the contractility of the heart improved.

## Discussion

Carcinomatous pericarditis is the process by which malignant pericardial effusion forms. It is an inflammatory condition of the pericardium that results from the extension of malignant cells to the pericardial sac. This inflammation can incite the accumulation of exudative fluid inside the pericardium creating a pericardial effusion. The pericardium is more commonly involved in secondary, or metastatic, neoplasms than primary neoplasms [6]. Lung, breast, hematological, and gastrointestinal carcinomas are the most common primary sites of metastatic involvement [7]. Of all the patients with metastatic neoplasms, 5%-20% have pericardial involvement; however, clinically significant pericardial disease is noticeably less common [8]. Pericardial effusions due to malignancy are typically larger and associated with worse outcomes compared to non-malignant effusions, and cardiac tamponade may occur in up to 50% of patients with malignant pericardial effusions [9].

Malignant pericardial effusions (MPEs) are a rare consequence of advanced malignancy but are correlated with significant morbidity and mortality. It bears a dismal prognosis as it represents the spread of malignant cells into the pericardium, which occurs in the setting of invasive local neoplasms or metastatic neoplastic dissemination. Several large retrospective studies [9-11] found that patients with previously diagnosed or newly detected malignancies had a mean survival of 4-6 months from the diagnosis of malignant pericardial effusion. However, non-malignant processes such as radiation therapy, chemotherapy, or infections were also discovered to be the source of pericardial effusion in approximately two-thirds of cancer patients [12]. Neoplastic cell positivity in pericardial fluid cytology is a separate indicator of poor prognosis [13,14].

Cardiac tamponade and other types of cardiogenic or obstructive shock might present with comparable symptoms, such as chest discomfort, palpitations, and shortness of breath. In more severe situations, patients may also exhibit syncope and altered mental state. The classic physical findings in cardiac tamponade are included in Beck's triad. Beck's triad consists of jugular vein distention (JVD), hypotension, and muffled heart sounds. Pulsus paradoxus, defined as a drop in systolic blood pressure (SBP) of more than 10 mmHg during inspiration, is another evidence that suggests pericardial effusion is producing cardiac tamponade. The Kussmaul sign, which is a paradoxical increase in jugular vein pressure (JVP) and pressure during inspiration, is another indication observed in tamponade [15]. The most common presenting symptom is dyspnea [16,17] as tamponade may reduce the heart's ability to pump blood efficiently, resulting in inadequate oxygen delivery.

The rapid accumulation of fluid in acute conditions without myocardium compensation may result in cardiac tamponade. This would manifest with chest pain in diseases such as acute aortic dissection or chest trauma [18,19]. However, in subacute conditions, pericardial effusion symptoms might range from no symptoms to generalized fatigue, to tamponade exacerbated by cardiac arrest. When there is a subacute or chronic pericardial effusion, the pericardial sac compensates with its elasticity until it achieves maximum tolerance [20].

With increasing pericardial effusion volume, the fluid will compress heart chambers and impair ventricular filling. High intrapericardial pressure will reduce venous return, resulting in a decrease in cardiac output. As a compensatory response, the patient may have tachycardia and tachypnea. A narrow pulse pressure would result from low cardiac output. Shortness of breath, tiredness, and leg edema are typical symptoms. While acute tamponade usually has a rapid decline, there is a fine line between significant stable pericardial effusion and subacute tamponade, which makes diagnosis difficult [3,21].

Because of inadequate filling, both right and left ventricular failure can occur as the fluid volume in the pericardial space grows due to epicardial or pericardial metastases or lymphatic obstruction. The volume of the MPE and the rate at which it is accumulating both determine the severity of symptoms; severe instances may manifest with shock and cardiac tamponade. Circumferential effusion can be brought on by 100 mL of pericardial fluid. Tamponade can be brought on by 300-600 mL of nonhemorrhagic pericardial fluid. However, if the fluid builds up rapidly, heart compression can happen at significantly lesser volumes [21].

Diagnosing tamponade based on clinical signs alone might prove difficult because they are neither sensitive nor specific. Based on the results of the physical examination and history, it can be suspected. The results of an ECG may also be useful, particularly if they reveal the classic tamponade ECG findings: low voltages or electrical alternans brought on by the heart swinging inside the fluid-filled pericardium; however, it is a rare ECG finding that is typically visible in severe situations. Sinus tachycardia is more likely to be evident on ECG. A chest X-ray is another useful technique since it can show an enlarged heart, which strongly implies pericardial effusion when contrasted to an earlier chest radiograph showing a normal cardiac profile. The gold standard imaging modality for tamponade diagnosis is echocardiography. It can identify pericardial effusions, quantify their extent, and determine whether they are impairing cardiac function (RV diastolic collapse, right atrial {RA} systolic collapse, and inferior vena cava {IVC} plethora). Several publications described clinicians (non-cardiologists) with limited point-of-care echocardiography training doing targeted echocardiograms to assess the presence of a substantial pericardial effusion [15].

POCUS can quickly and easily detect both sonographic tamponade and pericardial effusions [22]. Before a patient develops hypotension or exhibits any clinical symptoms or signs of tamponade, sonographic cardiac tamponade can be diagnosed. Sonographic tamponade is characterized by circumferential pericardial effusion, poor filling, and/or diastolic collapse of the right ventricle ("scalloping") brought on by increased intrapericardial pressure, resulting in decreased stroke volume and cardiac output [23,24].

Not only POCUS confirms the diagnosis, but also echocardiography can identify the optimal window for safely draining the fluid based on the amount and location of the fluid [24]. While Beck's triad is used to diagnose cardiac tamponade at the bedside, it is ineffective when a patient is in shock and hardly ever observed in practice. Beck's triad was discovered in a surgical population that experienced cardiac tamponade right away because of bleeding or trauma. Medical patients, on the other hand, are a population that experiences cardiac tamponade more gradually. The sensitivity under these circumstances can be as low as 20%, and the triad might not even be visible. So, two-dimensional echocardiography should be conducted since clinical examination findings are insufficiently sensitive [25]. The widespread use of POCUS has become an essential part of the treatment of critically ill patients, especially when time is of importance and a speedy diagnosis is required [26].

Malignant pericardial effusions (MPEs) have few treatment options, and they are rarely curative. Emergent pericardiocentesis is recommended in cardiac tamponade patients to avoid shock and death [27]. Most patients experience immediate symptom alleviation; however, the re-accumulation of the fluid may occur necessitating a repeat pericardiocentesis [28]. The malignant origin of the effusion is confirmed by pericardial fluid analysis, which also prevents hemodynamic compromise and recurrence. The treatment of the underlying malignancy improves outcome and prognosis, particularly when the effusion is linked directly to a locally invasive neoplasm [27,29]. Chemosensitivity or radiosensitive tumors, such as many lymphomas and previously untreated breast cancer, respond well to systemic chemotherapy or radiotherapy. Depending on the patient's overall course and reaction to therapy, the overall re-accumulation rates for both modalities are roughly 1/3 [30].

Regular pericardiocentesis can be used in preventing recurrences and injecting cytostatic and sclerosing chemicals intrapericardially with the goal of scarring the pericardium to the epicardium and preventing the MPE from re-accumulating. Several agents, such as doxycycline, minocycline, and bleomycin, have been investigated. About 70%-90% of patients achieve success (no re-accumulation at 30 days) [31,32]. Longer-term success rates, however, are unknown due to patient mortality. Less invasive surgical interventions include balloon pericardiotomies, subxiphoid pericardiostomies, and thoracoscopic pericardiostomies. More invasive surgical interventions include open thoracotomies with pericardial stripping. It is common to establish a pericardial "window," which involves the excision of a portion of the pericardium that allows continuous fluid drainage into the pleural cavity or externally [33-35]. According to studies [36-38], surgical interventions have minimal re-accumulation rates (less than 15% up to 10 months out).

## Conclusions

The course of treatment for MPEs is determined by the severity of the condition, the chance that the tumor will respond to antineoplastic therapies, and the patient's expected survival. It is advised to use a multidisciplinary approach when making decisions, incorporating information from the fields of cardiology, thoracic surgery, medical oncology, and radiation oncology. Patients with projected short survival periods (less than one month) might benefit from simple pericardiocentesis, especially if their MPE is not anticipated to recur in the remainder of their life span. A pericardiocentesis for symptom alleviation, followed by chemotherapy, may produce a long-lasting response in a symptomatic patient with no symptoms of tamponade and a chemotherapy-sensitive tumor, such as untreated breast cancer. Sclerosis or surgical decompression would be most beneficial for patients with longer prognoses (>1 month) whose MPEs are anticipated to re-accumulate; at this time, there is no conclusive data to suggest that one approach is superior to the other. Patients with very short prognoses and those who refuse more intrusive therapies should consider symptom-directed management without a specific intervention for the MPE.
